# Visible Light Active Natural Rutile Photocatalyst Obtained via Nano Milling

**DOI:** 10.3390/molecules30071600

**Published:** 2025-04-03

**Authors:** Kata Saszet, Enikő Eszter Almási, Ádám Rácz, Katalin Bohács, Milica Todea, Klára Hernádi, Zsolt Pap, Lucian Baia

**Affiliations:** 1 Nanostructured Materials and Bio-Nano-Interfaces Center, Interdisciplinary Research Institute on Bio-Nano-Sciences, Babeș-Bolyai University, Treboniu Laurian Street 42, RO-400271 Cluj-Napoca, Romania; kata.saszet@ubbcluj.ro (K.S.); almasieniko@geo.u-szeged (E.E.A.); todeam@phys.ubbcluj.ro (M.T.); 2 Faculty of Physics, Babeș-Bolyai University, M. Kogălniceanu Street 1, RO-400084 Cluj-Napoca, Romania; 3 Vulcano Research Group, Department of Mineralogy, Geochemistry and Petrology, University of Szeged, Egyetem Street 2, H-6722 Szeged, Hungary; 4 Institute of Raw Material Preparation and Environmental Processing, University of Miskolc, Egyetem Street 1, H-3515 Miskolc, Hungary; adam.racz@uni-miskolc.hu (Á.R.); katalin.bohacs@uni-miskolc.hu (K.B.); 5 Department of Molecular Sciences, Faculty of Medicine, Iuliu Haţieganu University of Medicine and Pharmacy, Victor Babeș Street 8, RO-400012 Cluj-Napoca, Romania; 6 Department of Applied and Environmental Chemistry, University of Szeged, Rerrich Béla Sqr. 1, H-6720 Szeged, Hungary; klara.hernadi@uni-miskolc.hu; 7 Institute of Physical Metallurgy, Metal Forming and Nanotechnology, University of Miskolc, Miskolc-Egyetemváros, C/1 108, H-3515 Miskolc, Hungary; 8 Laboratory for Advanced Materials and Applied Technologies, Institute of Research-Development-Innovation in Applied Natural Sciences, Babes-Bolyai University, Fântânele Street 30, RO-400294 Cluj-Napoca, Romania

**Keywords:** rutile, nano milling, ibuprofen, phenol, water treatment, photocatalysis

## Abstract

Natural rutile is a widely available titanium mineral which shows great potential as a photocatalyst for environmental remediation when processed correctly. Industries invest large sums in the transformation of the rutile mineral into pure, synthetic nano titania. Still, the present study proves that bare natural rutile with trace element content can also be applied as a photocatalyst, without harsh chemical interventions, simply by processing via nano grinding. Samples with different mean primary particle size values were obtained by wet stirred media milling, their compositional and structural properties were investigated, and their photocatalytic properties were evaluated under both visible- and UV-light illumination for the degradation of phenol and ibuprofen. By changing the grain size and the particle size distribution, and due to the doping effect of impurities present in the mineral, the band gap values of the samples and their photocatalytic activities changed as well. The nano milled rutile exhibited visible light photocatalytic activity, with a 33% degradation efficiency in the case of both phenol and ibuprofen, after 22 h of irradiation. The present study not only highlights the photocatalytic degradation of a pharmaceutical by natural rutile mineral, but its findings also suggest that ground nano rutile can function as an environmentally friendly photocatalyst, as it not only avoids the use of harmful chemicals typically employed in TiO_2_ synthesis but also offers a simpler, more cost-effective alternative for producing photocatalytic materials.

## 1. Introduction

Titania nanoparticles can be synthesized through various methods, which have been presented in the literature, such as sol-gel [1,2,3], hydrothermal [4]/solvothermal crystallization [5], direct oxidation [6], thermal decomposition [7,8], precipitation [9,10], hydrolysis [11,12], the sonochemical method [13,14], ball milling [15,16], chemical vapor deposition [17,18], spray pyrolysis [19,20], micelle- and inverse micelle-based methods [21,22], and microwave-induced crystallization [23,24]. Nano titania has a wide range of applications, including photocatalysis, a field that covers water splitting [25], sterilization [26], and pollutant degradation [27,28]. Titania is relatively inexpensive, has a high oxidation capacity, it is photostable, widely accessible and it is regarded non-toxic.

Titanium dioxide can be found in three different crystalline structures: rutile (tetragonal), anatase (tetragonal), and brookite (orthorhombic). Rutile is thermodynamically stable, while anatase is considered metastable [29]. Anatase can transform irreversibly into rutile, which process occurs around 600 °C [30], although depending on other conditions this temperature value can vary from 400 to 1200 °C [28,31,32]. Rutile is a more abundant mineral in nature, while anatase and brookite are quite rare. Rutile is widely distributed in metamorphic [33,34] and igneous rocks [35,36], soils [37], sedimentary deposits [38,39], and different sands [40].

Rutile is an important accessory mineral in metamorphic and magmatic rocks and occurs in sedimentary rocks as a detrital mineral. Natural rutile usually contains some additional minor elements, Fe, Cr, and Al, and several highly charged trace elements, such as Nb, Ta, Hf, Zr, Sb, W, Mo, Sn, Sb, W, U, and Sc [33,41,42,43]. The quantity of trace elements in rutile minerals varies: primary igneous rutile can have significant concentrations of Nb and Ta, which are widely used as geochemical fingerprints of geological processes [33].

The preparation of TiO_2_ nanoparticles utilizing natural rutile as raw material was previously attempted using spray pyrolysis [44], sonication [44], sol-gel methods, and ball milling [45] as a way of finding cheaper alternatives for TiO_2_ precursors. Taking the idea one step further, in a handful of studies, natural rutile was also directly involved in photocatalytic processes in various scenarios, but in each case, either the properties of the mineral were enhanced or the circumstances of the photodegradation tests were elevated. In one of the studies, rutile was used as a carrier for preparing synthetic TiO_2_ composites for methyl orange degradation [46], while other works used rutile samples processed by heating or quenching [47] or commercially purchased ones with unknown processing methods [48]. In three research studies, the ball milling or grinding method proved successful for natural photocatalyst preparation, but it must be mentioned that in two of these, the samples had a high content of FeTiO_3_ [49] or Fe_2_O_3_ [50], impacting the effectiveness of the rutile. In the third case for the photocatalytic degradation of methyl orange, besides rutile, H_2_O_2_ was added to the system and pH adjustments were made, which reportedly influenced the degradation efficiency [51]. Additionally, the decolorization of dye solutions was preferred [52], and no studies were found in the literature on the degradation of pharmaceuticals by natural rutile under visible light irradiation, except for the preliminary investigations of Almasi et al. whose findings served as a starting point of the present work [53].

TiO_2_ photocatalysts are widely used for the efficient degradation of dangerous organic contaminants such as phenol [54,55], pharmaceuticals (e.g., paracetamol, diclofenac, ibuprofen) [56,57], herbicides [58,59], and pesticides [60,61]. Many industries rely on phenol and phenolic compounds; therefore, these organic materials are often found in different wastewaters. These chemicals are highly water-soluble and dangerous for the environment and humans [62]. Another organic contaminant often found in natural waters is the pharmaceutical ibuprofen, which is a nonsteroidal anti-inflammatory drug widely used as a relieving medicine [63]. Although pharmaceutical residues are present in low levels in aquatic environments, there is a continuous input of these into the environment. They have impacted a wide range of aquatic species in various ways: abnormal development, lack of metamorphosis, and irregular behavior and reproduction [64,65]. As synthetic TiO_2_ photocatalysts are used for the degradation of both of these emerging contaminants—phenol and ibuprofen—it would make sense to also lead studies on natural rutile photocatalysts in this direction in addition to studying dye degradation, thereby tackling the oxidation of hazardous pollutants with naturally sourced solutions.

Considering that natural rutile is widely available and has a potential application in environmental remediation; by finding the right processing method, it can become a novel, cost-effective green photocatalyst. If the sample preparation of the natural mineral were reduced to mild purification methods and physical methods such as grinding, the process-related pollution would also decrease drastically. Not only it would eliminate a whole range of harmful precursors and agents used for the production of synthetic TiO_2_ [66] but it would also use a less complicated, shorter, and presumably cheaper process than the majority of multiple-step green synthesis methods or technologies [67,68,69].

In the present work, different natural rutile samples were prepared by the ball milling method using different grinding times. The samples were studied and their photocatalytic properties were determined. During the preparation, accompanying minerals were removed, and the milling process resulted in crystalline rutile samples with minor metallic impurities and significant fractions of nanoparticles. Considering the lack of studies on these contaminants, phenol and ibuprofen were selected as model organic pollutants to prove that it is possible to obtain a visible light active direct photocatalyst in a cost-effective way and avoiding chemical synthesis methods by applying mainly physical grinding processes.

## 2. Results

### 2.1. Structural and Morphological Characterization of the Ground Rutile

Before characterizing and involving it in any application, the excess minerals were removed to minimize their impact on the general properties of the ground rutile and on the morpho-structural properties of the processed rutile. The main component that had to be removed was Fe_2_O_3_, which was removed by applying 0.1 M oxalic acid aqueous solution under continuous ultrasonication. The minerals were further treated for 24 h in oxalic acid solution. The dilute solution of oxalic acid is preferred for the leaching of Fe_2_O_3_ as it is selective towards rutile [70,71]. It is considered a sustainable chemical and economically more feasible compared to inorganic acids, making the purification process cleaner and more environmentally friendly [72,73,74]. After the cleaning process, the rutile samples were washed with deionized water several times, resulting in the crystals shown in Figure S1. Thereafter, the rutile samples were ground as detailed in the experimental section.

**Figure 1:**
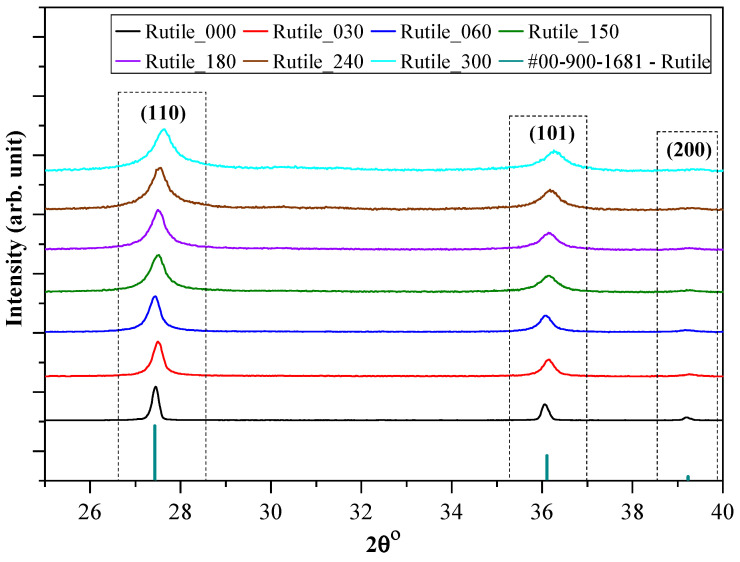
The XRD patterns of the rutile samples obtained after specific grinding times, showing the decrease in the primary crystallite size and pointing to the efficiency of the milling [53].

The first step in the characterization of the samples was to verify their crystal phase composition and primary crystallite size. The XRD pattern of the ground rutile (Figure 1) suggested a single mineral phase, rutile (TiO_2_), as expected. All the diffraction peaks were attributed to natural rutile (JCPDS card No. 00-900-1681), and the three strongest peaks were located at 27.44°, 36.08°, and 39.20°, corresponding to the (110), (101), and (200) crystallographic planes, respectively.

After the primary grinding process, the average particle size of the sample was determined based on the SEM micrographs and it was approximately 136 µm, while following the stirred media milling, the primary crystallite size significantly decreased with the grinding time. After 30 min, it decreased to 53.8 nm, while after 300 min, 20.1 nm was the average primary crystallite size (Table 1).

With the increase in grinding time, the calculated primary average crystallite size values were closer to the values obtained from the SEM micrographs. The structures and chemical compositions of the natural ground rutile samples were investigated using Raman spectroscopy; however, no significant changes were observed, as the Raman bands did not show any specific changes. On the Raman spectra of the rutile samples (Figure S2) the typical Raman bands for rutile (~250, 440, and 610 cm^−1^) were observable, which indicated the formation of a pure rutile TiO_2_ phase [33].

**Figure 2:**
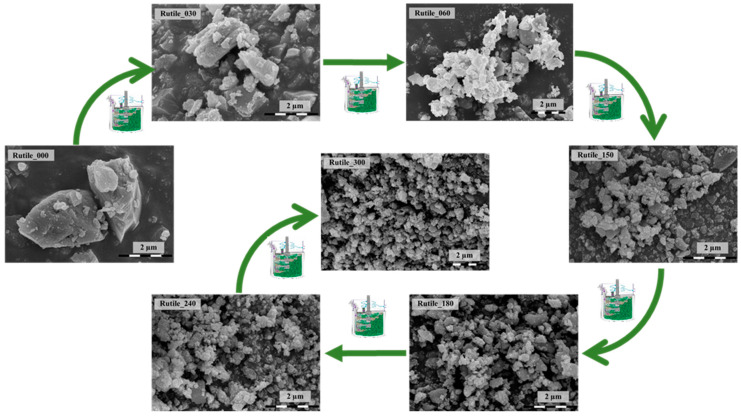
SEM micrographs of the samples obtained at different milling times, showing a uniform particle size distribution after 300 min.

The morphology of the ground rutile is shown in Figure 2. The surfaces of the samples were smooth and crack free; no protrusions were observed at all. The particle size showed a high degree of polydispersity. After 300 min of grinding, the rutile samples showed homogenous particles with non-specific geometries. The decrease in the rutile crystal size was also visible in the SEM micrographs. In the starting sample (Rutile_000), the following fractions were identified: ≤0.5 µm, 0.5–1 µm, 1–1.5 µm, 1.5–2 µm, 2–2.5 µm, 2.5–3 µm, and ≥3 µm particle sizes.

The sum of particles ≤0.5 µm in size indicated an increasing trend with prolonged milling duration. Interestingly, in the starting sample, the ≤0.5 µm particles were also present (17% was a large amount considering that the bare rutile was the natural sample itself), while after 30 min of grinding, the amount of ≤0.5 µm sized particles increased even more (to 31%), showing that a short grinding procedure was sufficient to increase the occurrence of nano-ranged particles.

**Figure 3:**
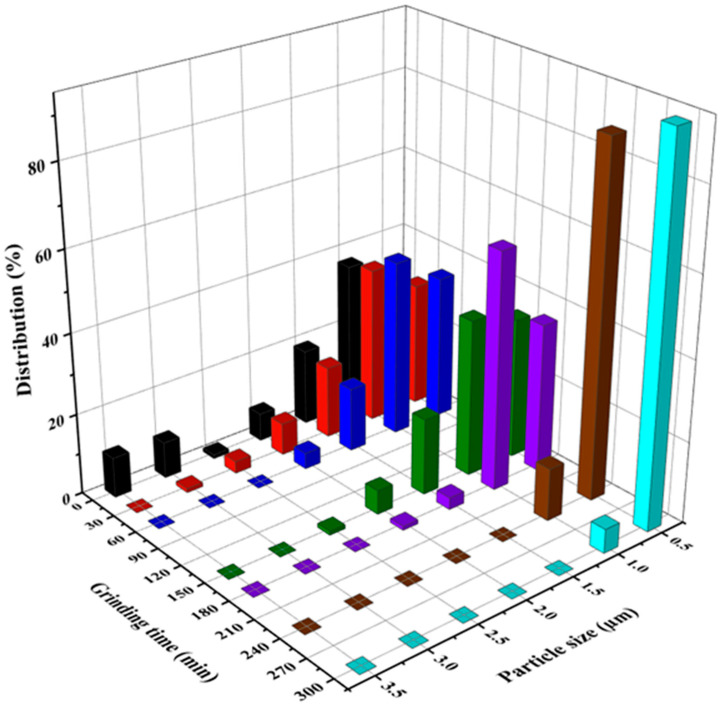
Particle size distribution histogram of ground natural rutile particles estimated from SEM micrographs.

After 60 min of continuous sample processing, a large quantity of rutile particles was under 1 µm (80%); among them, the amount of ≤0.5 µm sized particles increased further (36%). After 150 min, the ratio of 0.5–1 µm sized particles was 59%, while the quantity of ≤0.5 µm sized crystals remained nearly unchanged. After 180 min, the amount of ≤0.5 µm sized particles increased further (to 41%), and the amount of 0.5–1 µm sized particles decreased (to 39%). Following 240 min of grinding, the size of all the particles was under 1 µm, while the ratio of ≤0.5 µm sized particles was 87%. After the longest grinding period (300 min), the quantity of ≤0.5 µm sized crystals was even higher, achieving 94% abundance. All the investigated size ranges reached a maximum at a certain optimal grinding time (Figure 3).

**Figure 4:**
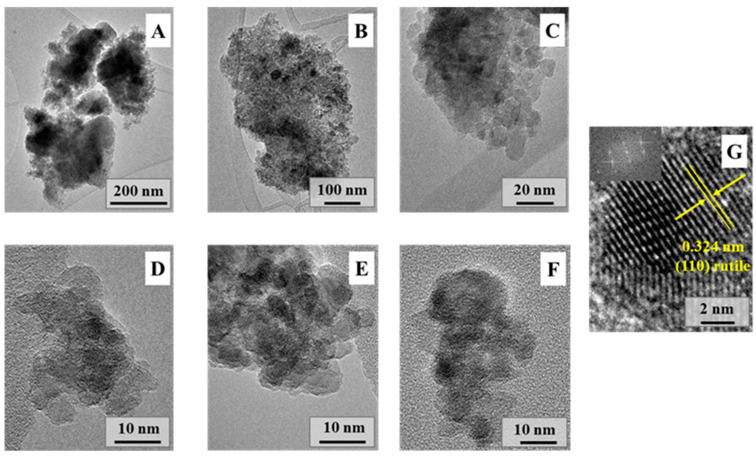
Transmission electron micrographs of the ground natural rutile (Rutile_300), showing the presence of differently sized aggregates (**A**–**F**). Rutile was also identified considering the interplanar distance (110) of rutile (**G**).

The morphological peculiarities of the ground natural rutile samples were also analyzed by TEM. Figure 4A–F show the micrographs of the ground natural rutile powders after 300 min. The aggregated rutile particles consisted of many nanoparticles of almost the same size; their average diameter was around 10 nm, and they were polyhedral in shape. While major aggregates were also observed (Figure 4A), smaller aggregates were dominant (Figure 4B–F). Moreover, it was found that the ground material was still crystalline (Figure 4G; the lattice fringes were visible even after 300 min of grinding), and no signs of recrystallization or any other kind of transformation were observed which could alter crystallinity related issues. This observation was also emphasized and demonstrated by Raman spectroscopy.

**Figure 5:**
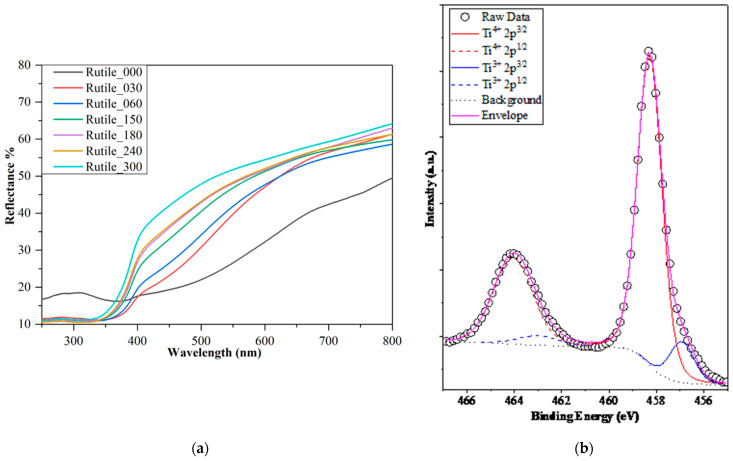
DRS and XPS spectra of the investigated samples, showing the change in band gap values (**a**) and the presence of Ti^3+^ in sample Rutile_300 (**b**).

The light absorption properties of the ground rutile samples were characterized by DRS measurements (Figure 5a), and band gap values (Table 1) were calculated using the Kubelka–Munk method. The band gaps of the ground rutile had energies of 2.6 ± 0.2 eV, narrower than synthetic rutile (3.03 eV) [75] but consistent with other natural rutile band gap values reported in the literature (2.7 eV [45] and 2.65 eV [51]). The surprisingly low values can be explained by the presence of additional minor elements in the mineral. These impurities act as dopants, modifying the optical properties of the pure rutile.

One of the various techniques, which can be used to alter the band gap of TiO_2_ is elemental doping [76]. The doping elements used for synthetic TiO_2_ presented in the literature are metal ions (predominantly transition metals), Cu, Co, Ni, Cr, Mn, Mo, Nb, V, Fe, etc., and non-metal elements as well, such as N, S, C, B, P, I, and F [77,78]. These dopants have a positive effect in most cases, increasing the photocatalytic activity [79,80], but exceptions can also be found in the literature [81]. Moreover, there are already cases documented in the literature where the optical properties of natural rutile were enhanced by V^5+^, Fe^3+^, Nb^5+^, and other metallic impurities present in the sample [34,45,47,51]. According to the performed XRF measurements, the ground rutile sample Rutile-180 investigated in the present research had higher concentration of Hf (4165 ppm), Nb (2475 ppm), Nd (1092 ppm), Zr (57,630 ppm), and W (230 ppm), than the rutile sample which received the highest quantity (37,012.6 kJ/kg) of specific grinding energy (Rutile_300, Table 1). The trace elements detected in this sample namely Hf (1487 ppm), Nb (506 ppm), Nd (506 ppm), and Zr (19,210 ppm), were present in a significantly decreased amount [53]. This change in the concentration of accompanying elements may have several causes, one of them being their slow dissolution in water during the milling process. The appearance of the above-listed metals in the rutile samples is not a surprise, as these tend to occur simultaneously with Ti. The list of other elements detected by XRF is presented in Table S1. All five of the prior impurities are reported to be dopants of TiO_2_, but only Nb, Nd, Zr, and W dopants reduce unequivocally the band gap of pure TiO_2_ [82,83,84,85,86,87,88,89,90]. The effect of Hf doping on the TiO_2_ band gap is not clear: based on the literature, it can lead both to narrowing [91,92] and enlarging [93,94], and there is also a lack of studies of Hf-doped TiO_2_ applied in photocatalysis. On the other hand, doping with Nb, Nd, Zr, or W leads to an extended absorption range in the visible light and higher photocatalytic efficiency [82,83,85,86,89].

A gradual band gap change in the ground samples was induced by the milling processes. The initial band gap value (2.4 eV) rose after 300 min of grinding to 2.8 eV. While the band gap values increased with the grinding time, the average primary crystallite size was reduced concomitantly. One of the explanations of the gradually wider band gap of the samples could be the size effect phenomenon induced by the small fraction of particles below 10 nm. Although the smallest particles could be identified from the SEM micrographs as particles <0.5 μm, the size distribution of this fraction was wide. Based on the TEM micrographs, nanoparticles with an average size of 10 nm and below were also present in the samples in higher and higher percentages as the grinding process advanced, resulting in the widening of the observed band gaps. The increase in the band gap energy with diminishing primary crystallite size is a known phenomenon, documented also in the case of TiO_2_ nanoparticles below 10 nm [95,96].

Examining the measured DRS spectra (Figure 5a), besides the blue shift of the adsorption edge, one more spectral characteristic is observable, namely, the broadness of the non-linear region, which indicates the possible presence of impurities or defects in the crystal lattice. To further investigate these possibilities and point out energetic disorders in the materials, their Urbach energy was determined [97,98]. While the Urbach energy of the sample with the least grinding was 386 meV, the values declined with the grinding time; the last one measured 285 meV after 300 min of milling (Table 1), which approaches the values reported for pure, synthetic forms of TiO_2_ [99]. The decrease in the Urbach energy values suggests the enhancement of the materials’ quality and ordering after ball milling, which is not a typically expected finding, as grinding is known to more likely induce stress in the material [100]. Nevertheless, the findings of other investigation methods, such as the unshifted peaks in the Raman spectra, corroborate the lack of strain in the crystal lattices. Another factor which could influence the Urbach energy values of a material is the presence of impurities or dopants [101,102,103], which can be found in abundance in natural rutile. Interestingly, the changes in the Urbach energy values complemented the trend of the band gap and grinding time, and both can be explained by a possible change in the quantity of impurities. As the milling of the samples advanced, the band gap values experienced a slight rise while the Urbach energy values declined. Each energetic value change suggested that less and less impurities, i.e., dopants, were present in the mineral composition. The lower quantity of accompanying elements after a longer grinding time was already observed in the results of the XRF measurements; thus, besides the possibility of size effect, another phenomenon influencing the small and gradual increase in the band gap values could be the slow elimination of impurities from the mineral during ball milling.

However, it should be noted that photocatalytic reactions occur on the surface of the catalyst particles; therefore, the surface quality of the individual nanocrystals should be checked and that is why XPS was applied. Rutile-300 was chosen for this analysis, as it had the smallest primary crystallite size and, thus, its surface element concentration values should be maximized.

During the XPS investigations, the following elements were successfully identified (besides the expected C, O, and Ti): Zr, Nd, Sn, and Cr. Hf, Nb, W, and Ta were also detected, but in extremely low amounts (Figure S3). In the case of the first element group, the oxidation states and the concentrations of these elements on the surface were estimable, while in case of the second group, only their presence could be confirmed. The carbon content found in the sample was a usual value, as the surface of any material is immediately occupied by organic molecules present in the air [104]. However, one major surface feature was observed, namely, the ground samples contained Ti^3+^ centers, as shown in Figure 5b. Moreover, the presence of Ti^3+^ was also demonstrated in the rutile crystals that originated from chloritoid-grade metapelites [34]. This is quite encouraging, as Ti^3+^ center formation usually demands a reductive synthesis atmosphere which is not easy to handle or to manipulate if a lab synthesis is carried out. As the grinding process does not occur under a reductive atmosphere, it can be safely presumed that the Ti^3+^ identified in the mineral is of natural origin.

It is widely known that the appearance of such entities in the structure of the surface layers could lead to enhanced photocatalytic activity [105] by generating additional OH radicals [106]. In this sample, the amount of Ti^3+^ was 11.2% of the total amount of Ti atoms. This number is, however, relatively high and could lead to enhanced recombination. Nevertheless, besides this element, others were also detected, and as mentioned before, and each of them probably contributed to the overall photocatalytic activity.

### 2.2. Photocatalytic Performance of the Samples

The ground rutile samples were first applied in UV light-assisted photocatalytic degradation tests of phenol. After a 2 h irradiation, all the rutile samples showed negligible degradation efficiencies. The most noticeable difference in the phenol concentration was observed in the case of the Rutile_180 sample, which still only achieved a 2.7% decomposition (Figure S4). Taking into consideration the effects of doping discussed previously, namely the expansion of the absorption edge into the visible region, it was expected to achieve a higher degradation efficiency under visible light irradiation and perhaps a hindered one in UV-assisted degradation. As the degree of photolysis of phenol in similar experimental conditions can rise up to 4% [107,108], the photolysis test was also performed to weigh its influence on the photocatalytic degradation results. The phenol solution showed a 0.6% concentration decrease under UV irradiation (Figure S4) after 2 h, which falls in the same category as the measured reduced photocatalytic degradation values. Considering the above points, the UV light-assisted photocatalytic degradation tests were stopped after 2 h, and the focus was shifted to the tests performed under visible light irradiation. The decision was made based on the previously revealed compositional, structural, and optical properties of the samples and the common objective of the field: the development of a visible light active, efficient photocatalyst that uses a more sustainable source of energy.

**Figure 6:**
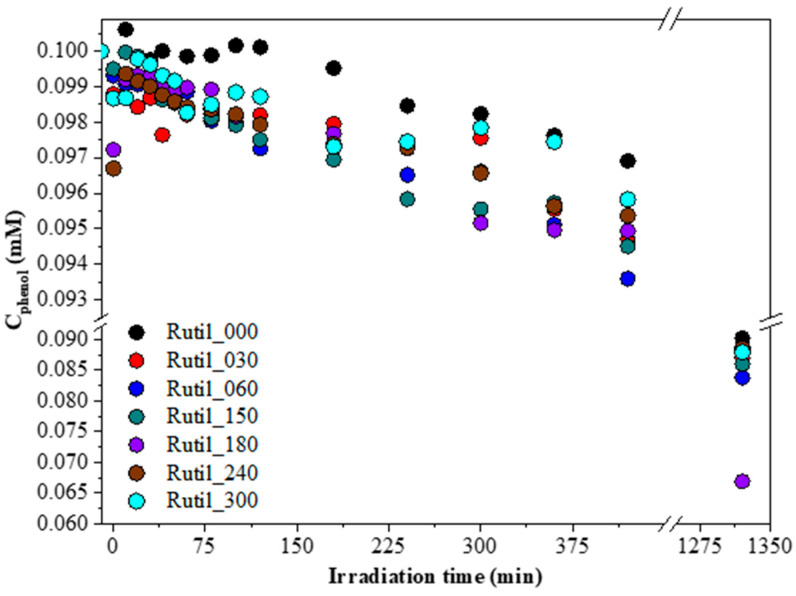
Photocatalytic degradation curves of phenol under visible light irradiation (c_phenol_ = 0.1 mM) for natural rutile samples.

In case of the visible light-driven experiments, the situation was different. Figure 6 shows the obtained phenol degradation curves under visible light irradiation. In the first instance, it was expected that, similar to the UV-assisted tests, the degradation rate would be minuscule (that is why the duration of the degradation experiment was chosen to be 22 h). Nevertheless, the highest achieved degradation yield was 33% (sample Rutile_180), which is a remarkable result considering that no chemical synthesis processes were used to obtain these catalysts. Moreover, the degradation procedure was confirmed by HPLC, and the two main oxidation byproducts of phenol (hydroquinone and pyrocatechol) were detected.

To exclude further factors that could influence the degradation rate of phenol, a photolysis test was performed. The direct photolysis of phenol under visible light irradiation was negligible, as phenol has its absorption maximum in UV light (λ_max_ = 270 nm) and it is stable in the visible light region [109]. During the 22 h irradiation test, the concentration declined by less than 2% (Figure S5), which is an order of magnitude smaller than the best measured 33% degradation yield and does not significantly affect the degradation efficiency.

Interestingly, even the sample that was pre-ground for nano milling (Rutile_000) showed a 10% efficiency of phenol degradation. This finding indicated that the photocatalytic activity of the samples might be influenced also by their specific surface area. To further investigate this correlation, the specific surface area of each sample was measured, and their surface normalized photoactivity was also assessed (Figure S6).

**Figure 7:**
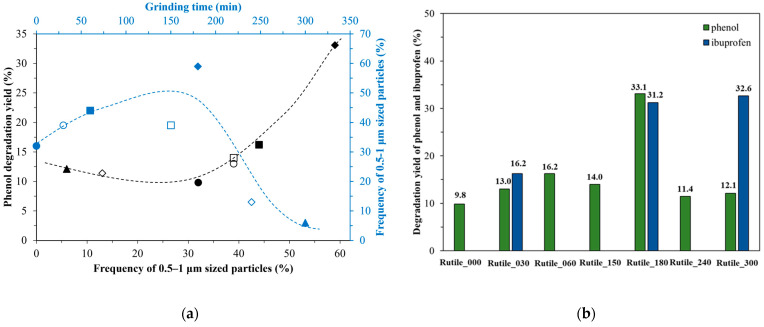
The phenol degradation dependency from the frequency of 0.5–1 μm sized particles (black series) and their formation at different grinding times (blue series). The samples were individually coded as follows: ●●—Rutile_000, **○○**—Rutile_030, ■■—Rutile_060, **□□**—Rutile_150, ♦♦—Rutile_180, ◊◊—Rutile_240, ▲▲—Rutile_300. The used trendlines are for visualisation purposes and do not represent any kind of mathematical trend (**a**). The individual photocatalytic performance under visible light irradiation of the nano milled rutile samples (**b**).

The specific surface area of ground rutile increased with the applied grinding time from 1 m^2^∙g^−1^ to 80 m^2^∙g^−1^ (Table 1). As expected, it followed the trends observed in the evaluation of the diffraction patterns and the different electron micrographs. Interestingly, the surface normalized activity decreased with the duration of the nano milling (Figure S6). The sample which received only pre-grinding (Rutile_000) possessed the highest activity (983.6∙10^−5^ mM/m^2^ compared to sample Rutile_300, which showed an activity value of 19.4∙10^−5^ mM/m^2^). This demonstrates that the trace elements detected and discussed previously were slowly leaking out during the milling process, while the lack of activity-conferring elements was compensated by the higher specific surface area values. Although, the surface area overcompensated the loss of the dopant elements, which justifies the nano milling. While the surface normalized photoactivity can be useful to evaluate the surface quality of the samples, for real-life applications the absolute activity is taken into account. The best samples for visible light phenol degradation were Rutile_60, Rutile_180, and Rutile_300. The activity differences between these samples may depend on several factors; however, there are two major issues. The first is the presence of Ti^3+,^ and the second is the presence of 0.5–1 μm sized particles. The latter demonstrates a clear positive correlation, indicating that as the frequency of the specified particles increases, the photocatalytic activity correspondingly enhances (Figure 7a). This explains the high activity of Rutile_180, but still more scientific data is needed to explain how nano grinding influences the properties of minerals. Three samples were chosen from the grinding series to evaluate their photoactivity towards a real pollutant, ibuprofen. These samples were the samples with the shortest and the longest grinding times, Rutile_30 and Rutile_300, and Rutile_180, the sample with the highest degradation efficiency in the case of phenol. Similar to the case of phenol, the samples were not active in UV-A light, only in visible light. Every chosen sample showed photocatalytic activity under visible light degradation, the highest being achieved by Rutile_300 (32.6% ibuprofen degradation), while Rutile_30 achieved only 16.2% degradation (Figure 7b). It seems that the photocatalytic activity towards ibuprofen increased with the grinding times, and, consequently, with the decrease in the particle size. The trend of lower particle size and higher activity was already described in the literature [110]. Ibuprofen, similar to phenol, is photostable under visible light, as its absorption maximum is in the UV region (λ_max_ ≈ 222 nm) [111]; thus, the visible light-assisted direct photolysis was disregarded.

### 2.3. Comparison of Production Method of Natural and Synthetic TiO_2_

Besides efficiency, many factors influence the feasibility of using a material in the field of photocatalysis. One of these factors is the cost-effectiveness of the choice—and it is a very important aspect to consider. To compare the economic viability of a photocatalyst gained solely through mineral processing to a synthetic one, their production processes should be considered in detail.

**Figure 8:**
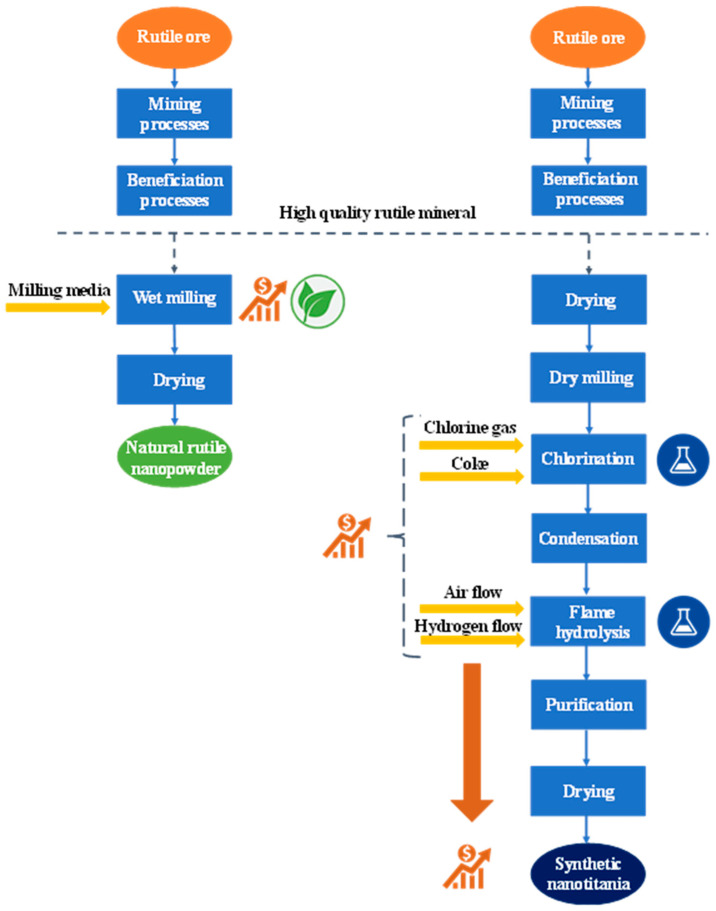
Schematic comparison of natural rutile processing and synthetic TiO_2_ production methods.

Figure 8 follows the steps of both processes, highlighting the resource and cost differences. The wet milling method chosen for the processing of natural rutile has a better grinding efficiency than dry milling, results in finer particles, has better heat dissipation, and it is also a greener method compared to any other chemical synthesis, but to achieve all this, it requires sophisticated equipment and can be an energy-intensive process [112].

To estimate the cost of production of the natural rutile photocatalyst, the price of the raw material and similar conditions were considered, as detailed in the Materials and Methods Section. A 180 min grinding time in a Netzsch MiniCer stirred media mill (Netzsch GmbH, Selb, Germany) was followed by a 24 h drying cycle in a drying oven. Under these conditions, the production costs of the natural rutile photocatalyst were estimated to be 9.93 €/kg (calculations are provided in Supplementary Information).

Although using the more costly wet milling method is an argument against the use of the natural rutile photocatalyst, besides drying, this is the last step of its processing and results in a ready-to-use natural photocatalyst. However, for the synthetic nano titania, this is only the preparation phase of the precursor.

The most well-known commercial TiO_2_ photocatalyst is Evonik AEROXIDE^®^ TiO_2_ P25 (Evonik Industries AG, Essen, Germany), which is produced via the patented Aerosil process [113]. This method is known to be a flame hydrolysis process which uses TiCl_4_ as precursor [114]. TiCl_4_ is most commonly acquired from processed rutile mineral through a chlorination reaction [115]. Each step of this synthesis chain introduces additional chemicals and results in byproducts, not only raising the costs but also the environmentally harmful aspects of the method. The price of the as-produced high purity TiO_2_ P25 is around 265 €/kg, depending on market fluctuations [116].

The comparison of the production of synthetic TiO_2_ P25 and natural rutile powder was made on a laboratory scale for a simple reason: as there is no commonly known process in the industry for the production of a natural rutile photocatalyst as described in this study, the data necessary for a correct scale-up of the costs are not available.

However, it is generally true that on an industrial scale, the cost of production is lower for various reasons, such as the bulk purchasing of raw materials or better equipment utilization. This explains the low price of synthetic TiO_2_ P25 bought in bulk (tons) on the market—around 2 €/kg [117]—but also suggests that natural rutile processing on an industrial scale would result in a lower unit price than the one considered in the calculations of the present study.

To correlate the cost of both photocatalysts with their efficiency in photodegradation, as a last step, the cost of degradation of 1 mmol phenol/hour was calculated for each. For this, the 33.1% efficiency of the Rutile_180 sample during the 22 h irradiation time was compared to the 21.1% degradation efficiency of phenol using TiO_2_ P25, measured under the same circumstances but during a 2 h photocatalytic run (Figure S4). The results were not surprising: while the synthetic nano titania costs roughly 25 € for each mmol of degraded phenol/hour, the cost of phenol degradation in the case of natural rutile was estimated to be almost four times lower (6.6 €/(mmol/h)).

## 3. Materials and Methods

### 3.1. Materials

Natural rutile (TiO_2_) from Brazil was used as the raw starting material. The rutile mineral had a deep grey color with submetallic luster, and 3 to 5 cm large grains. The crystals were short prismatic ones. On the surface of the rutile crystals cracks were observed, along with brownish iron(III) oxide. For the iron(III) oxide removal an aqueous oxalic acid (Sigma-Aldrich, Merck KGaA, Darmstadt, Germany, >99.0% purity) solution was used. The photocatalytic efficiencies were determined using aqueous solutions of phenol (Spectrum Chemical Manufacturing Corp., Gardena, CA, USA, analytical grade) and ibuprofen (Fluka^TM^, Merck KGaA, Darmstadt, Germany, >99.0% purity) as the water contaminant. As a reference, commercial photocatalyst Evonik AEROXIDE^®^ TiO_2_ P25 (Evonik Industries AG, Essen, Germany) was used.

### 3.2. Applied Methods

In the first step, the rutile samples were ground to a particle size below 106 µm in a Retsch planetary ball mill. The name Rutile-000 was given to the as-gained sample, which was used afterwards as a feed for the nano grinding. For the natural rutile grinding, a stirred media mill (Netzsch MiniCer, Netzsch GmbH, Selb, Germany) was used in continuous closed-circuit mode. The liners of the grinding chamber and the stirrer rotors were made of ZrO (zirconia). The circumferential tip speed of the rotor was 9.3 ms^−1^ during the experiments. Yttrium-stabilized zirconium oxide (ZY Premium, Sigmund Lindner GmbH, Warmensteinach, Germany) grinding media was applied during grinding. The filling ratio of the grinding ball in the milling chamber was 70 *v*/*v*%. Mass concentration of the suspension was 0.2 *m*/*m*%. Samples were taken after 30-, 60-, 150-, 180-, 240-, and 300-min grinding times (the samples were coded based on the grinding times, e.g., Rutile_030 denotes the sample milled for 30 min). Until 180 min grinding time, 0.8–1.0 mm grinding media was used, then it was changed to a smaller one (0.6–0.8 mm) for more effective grinding. Suspension samples were taken from the outflow of the pipe in which the suspension re-entered the stirrer vessel. The volumetric flow rate of the pump was 2.5 × 10^−5^ m^3^s^−1^. The power draw of the mill was measured by the Netzsch measuring system, and data were registered in the computer. In this way, the specific grinding energy could be calculated.

The crystal phase identification and the primary mean crystallite size values were determined by a Rigaku MiniFlex II diffractometer (Rigaku Corporation, Tokyo, Japan) equipped with a graphite monochromator and functioning with Cu-K_α_ radiation (λ = 0.15406 nm, 30 kV, 15 mA). The scanning speed was 1(2θ°) min^−1^. The XRD measurements were recorded in the 2θ° range from 20–40°. The mean primary crystal size values were calculated using the Scherrer equation [118].

The particle size, microstructure, and morphology of the minerals were analyzed using a cold field-emission scanning electron microscope (SEM, Hitachi S-4700 Type II, accelerating voltage: 10 kV; Hitachi Ltd., London, UK). The samples for SEM measurements were attached to a carbon adhesive pad, which was fixed to an aluminum sample holder.

Transmission Electron Microscopy (TEM) micrographs were obtained to analyze the morphology of the nanoparticles with a FEI Tecnai G2 20 X-TWIN (FEI Company, Hillsboro, OR, USA) instrument operating at an accelerating voltage of 200 kV.

The crystalline structure of the rutile mineral was determined by Thermo Scientific DXR confocal Raman microscope (Thermo Fisher Scientific Inc., Waltham, MA, USA), equipped with a diode-pumped frequency-doubled Nd:YAG laser (532 nm laser, 8 mW laser power, 10× objective lens, spot size of approximately 1 µm). The acquired spectra were recorded at 2 cm^−1^, and a 25 µm pinhole confocal aperture was used for each measurement.

A JASCO-V650 (Jasco Corporation, Wien, Austria) diode array computer controlled (SpectraManager Software v2.8) spectrophotometer with an integration sphere (ILV-724) was used to measure the Diffuse Reflectance (DR) spectra of the samples (λ = 250–800 nm). To obtain the band gap energy values, the well-known Kubelka–Munk approach was applied [119].

The trace element content of the samples was measured with a Horiba Jobin Yvon XGT-5000 X-ray fluorescent spectrometer (Horiba Ltd., Kyoto, Japan), equipped with Rh X-ray source. The records were made at 30 kV excitation voltage, 0.5 mA anode current, and 1000 s measuring time.

X-ray photoelectron spectra of rutile samples were performed on a SPECS instrument equipped with a PHOIBOS 150 MCD 9 hemispherical electron energy analyzer operated in the FAT mode (Specs GmbH, Berlin, Germany). The system employed a monochromatic Al-K source (1486.6 eV) at 14 kV and 20 mA. Samples were fixed on a double-sided adhesive carbon tape and care was taken to ensure that the sample particles covered the tape. Experiments were performed by operating the X-ray source with a power of 200 W, while the pressure in the analyzer chamber was in the range of 10^−9^–10^−10^ mbar. The binding energy scale was charge referenced to the C1s at 284.6 eV. High resolution spectra of all the detected elements were obtained using an analyzer pass energy of 20 eV, in steps of 0.05 eV, for the analyzed samples. Analysis of the obtained data was carried out with Casa XPS software v2.3.19PR1.0. All peaks were deconvoluted using Shirley background and Lorentzian–Gaussian line shapes. The applied value of the Gaussian–Lorentzian ratio was 30.

The specific surface areas of the ground rutile samples were determined by nitrogen adsorption at 77 K using a BELCAT-A device (Microtrac Retsch GmbH, Duesseldorf, Germany). The specific surface area was calculated via the BET method.

### 3.3. The Photocatalytic Activity Determination

The photocatalytic activity of the natural rutile was determined by the photodegradation of phenol and ibuprofen under UV and visible light irradiation. An aliquot of 100 mL of the photocatalyst dispersion (c_rutile_ = 1.0 g/L) containing phenol (c_phenol_ = 0.1 mM) or ibuprofen (c_ibuprofen_ =0.1 mM) was used in the experiments. The photoreactor used for the visible light experiments was an open glass tube with double walls, equipped with an OSRAM metal halide lamp (Osram Sylvania Inc., Wilmington, MA, USA; Power Star HCl-TC 75W/WDL type) and surrounded by a thermostating jacket maintained at 25.0 °C. In the thermostating jacket, an aqueous NaNO_2_ solution (1 M, Molar Chemicals Kft., Halasztelek, Hungary; min. 99.13%) was circulated to absorb UV photons (λ< 400 nm), which ensured that only visible light irradiation reached the suspension. The irradiance measured in the reactor position was 18 mW/cm^2^. The UV photoreactor was irradiated by six fluorescent lamps (Vilber–Lourmat T-6L UV-A, 6 W power, radiation maximum at 365 nm; Vilber Company, Paris, France) and the thermostating agent was pure water (T = 25 °C). Prior to the photocatalytic experiments, the suspensions were sonicated in the dark for 15 min, then they were vigorously stirred by a magnetic stirrer during the measurements. Dissolved oxygen concentration was maintained constant by bubbling air through the reactor. Samples of 1.5 mL were taken out at specific times (0, 10, 20, 30, 40, 50, 60, 80, 100, 120, 180, 240, 300, 360, 420, and 1320 min), and they were centrifuged for 3 min (at 13,400 rpm) and filtered with a Whatman Anotop Syringe Filter (Whatman plc., Maidstone, UK).

The duration of the photocatalytic experiments was 2 h (120 min) in the case of UV irradiation and 22 h (1320 min) in the case of visible light irradiation. In each case, the level of the suspension was marked on the photoreactor and the potential evaporation of the water content was carefully monitored during the lengthy photocatalytic tests. To maintain the accuracy and consistency of the photodegradation experiments under visible light irradiation, after 360 min, distilled water was gradually added, if necessary, to replenish the evaporated solution (1–2.5% of the total volume after 360 min). This process was repeated at the end of the photocatalytic experiments.

The phenol concentration was determined with High Performance Liquid Chromatography using a Merck-Hitachi L-7100 low-pressure gradient pump (Merck, Darmstadt, Germany) equipped with a Merck-Hitachi L-4250 UV–Vis detector (Merck, Darmstadt, Germany) and a Lichrospher RP 18 column with a methanol/water mixture (50:50) as eluent. The detection wavelength for phenol was λ = 210 nm. The concentration of ibuprofen was determined using an Agilent 8453 UV-Vis spectrophotometer (Agilent Technologies GmbH, Waldbronn, Germany) and a 0.2 cm cuvette, and the detection wavelength was λ = 222 nm.

The photocatalytic activity of TiO_2_ P25 was also evaluated under visible light irradiation for phenol (c_phenol_ = 0.1 mM) degradation, and it was used as a reference. The duration of this photocatalytic run was 2 h. Other parameters remained the same as described above in the case of natural rutile.

## 4. Conclusions

The photocatalytic performances of ground natural rutile samples were examined for phenol and ibuprofen degradation under visible light and UV light irradiation. The minerals were ground for 30, 60, 150, 180, 240, and 300 min. Due to the milling process, the particle size, particle size distribution, surface area, band gap energy, and photocatalytic activity changed significantly. The chemical composition of the samples was also evaluated, and it showed the presence of several dopant elements, while on the surface of the samples, the presence of Ti^3+^ centers was demonstrated. Both characteristics shed light on the enhanced activity of the minerals under visible light irradiation.

The sample Rutile_180 was the most efficient sample for phenol degradation under visible light irradiation. It decomposed 33.1% of the phenol in 22 h. The same sample, Rutile_180, and the sample Rutile_300 also showed good photocatalytic activity for ibuprofen degradation (31.2% and 32.6%, respectively). Thus, the present study highlights the degradation of a pharmaceutical by a rutile mineral photocatalyst under visible light irradiation. We show that the presence of 0.5–1 μm sized particles was the key for the photoactivity towards phenol, while in case of ibuprofen, the higher grinding times resulted in higher activity. In comparison, the UV activity under the applied conditions was insignificant and was more comparable in size with the photolysis values.

The present study proves that rutile, despite being a simple natural mineral, can be activated under visible light irradiation for the degradation of organic contaminants and pharmaceuticals. In addition, on the laboratory scale, its production is estimated to be four times more cost-efficient than the classical chemical synthesis methods applied for nano titania production. Although there is a need for further research in order to accomplish a viable scale-up of the nano milling production method, it has great potential in the market of photocatalysts prepared with environmentally friendly processing methods.

## Figures and Tables

**Table 1:** Morpho-structural properties and photocatalytic activities of the investigated rutile samples [53].

Sample	Grinding Time (min)	Median Crystal Size (µm) from SEM Micrograph	Grinding Energy (kJ/kW)	Bandgap Energy (eV)	Urbach Energy (meV)	Primary Crystallite Size (nm)	Specific Surface Area (m^2^∙g^−1^)	Phenol Degradation (%)	Surface Normalized Transformed Phenol Concentration (mM/m^2^)∙10^−5^	Ibuprofen Degradation (%)	Surface Normalized Ibuprofen Degradation (mM/m^2^) 10^−5^
Rutile_000	0	1.9	0.00	-	-	132.0	1	9.8	983.6	-	-
Rutile_030	30	0.9	3782.7	2.42	386	53.8	11	13.0	118.3	16.2	7.61
Rutile_060	60	0.8	7565.3	2.47	369	44.1	20	16.2	81.2	-	-
Rutile_150	150	0.7	18,913.3	2.62	333	31.3	41	14.0	34.1	-	-
Rutile_180	180	0.7	22,696	2.68	316	31.4	49	33.1	67.6	31.2	1.40
Rutile_240	240	0.6	29,854.3	2.71	313	25.3	59	11.4	19.4	-	-
Rutile_300	300	0.5	37,012.6	2.78	285	20.1	80	12.1	15.1	32.6	0.84

## Data Availability

The original contributions presented in this study are included in the article/Supplementary Materials. Further inquiries can be directed to the corresponding author.

## Supplementary Materials

The following supporting information can be downloaded at: https://www.mdpi.com/article/10.3390/molecules30071600/s1. Figure S1: Photographs of the short prismatic rutile crystals from Brazil (the scale bar is 1 cm). Figure S2: The Raman spectra of the ground rutile, showing insignificant changes during the grinding process. Figure S3: The XPS survey spectrum of the ground Rutile_300 sample. Figure S4: Phenol photolysis and photocatalytic degradation of the Rutile_180 sample under UV light irradiation. Figure S5: Phenol photolysis under visible light irradiation. Figure S6: The surface normalized photocatalytic activity of ground natural rutile samples for the degradation of phenol. Figure S7. Photocatalytic degradation curve of phenol (c_phenol_ = 0.1 mM) under visible light irradiation in the presence of commercial Evonik Aeroxide TiO_2_ P25. Table S1: Trace element concentration of ground natural rutile samples by XRF/XPS analysis. 3. Cost-benefit calculations. Table S2: List of calculated data, step by step, to correlate the cost and the efficiency of the natural and synthetic photocatalysts. References [53,120,121,122,123,124] are cited in the supplementary materials.
