# Supplementary material for: Visible Light Active Natural Rutile Photocatalyst Obtained via Nano Milling
Source: Molecules. 2025 Apr 3;30(7):1600. doi: 10.3390/molecules30071600 (PMC11990522; doi:10.3390/molecules30071600)
Supplement: Supplementary file 1 [file molecules-30-01600-s001.zip › molecules-3493507-supplementary.pdf]

Supplementary materials

Visible-light active natural rutile photocatalyst obtained *via* nano milling

Kata Saszet [1,2†], Enikő Eszter Almási [1,3†], Ádám Rácz [4], Katalin Bohács [4], Milica Todea[1,5], Klára Hernádi [6,7], Zsolt Pap[1,6,8*], and Lucian Baia [1,2,8*]

1 Nanostructured Materials and Bio-Nano-Interfaces Center, Interdisciplinary Research Institute on Bio-Nano-Sciences, Babeș-Bolyai University, Treboniu Laurian Street 42, RO-400271 Cluj-Napoca, Romania;
2 Faculty of Physics, Babeș-Bolyai University, M. Kogălniceanu Street 1, RO-400084 Cluj-Napoca, Romania;
3 Vulcano Research Group, Department of Mineralogy, Geochemistry and Petrology, University of Szeged, Egyetem Street 2, H-6722 Szeged, Hungary;
4 Institute of Raw Material Preparation and Environmental Processing, University of Miskolc, Egyetem Street 1, H-3515 Miskolc, Hungary;
5 Department of Molecular Sciences, Faculty of Medicine, Iuliu Haţieganu University of Medicine and Pharmacy, Victor Babeș Street 8, RO–400012 Cluj-Napoca, Romania;
6 Department of Applied and Environmental Chemistry, University of Szeged, Rerrich Béla Sqr. 1, H-6720 Szeged, Hungary;
7 Institute of Physical Metallurgy, Metal Forming and Nanotechnology, University of Miskolc, Miskolc-Egyetemváros, C/1 108, HU-3515 Miskolc, Hungary;
8 Laboratory for Advanced Materials and Applied Technologies, Institute of Research-Development-Innovation in Applied Natural Sciences, Babes-Bolyai University, Fântânele Street 30, RO-400294, Cluj-Napoca, Romania;

† These authors contributed equally to this work.
* Correspondence: zsolt.pap@ubbcluj.ro; pzsolt@chem.u-szeged.hu; lucian.baia@ubbcluj.ro;

1. Figures

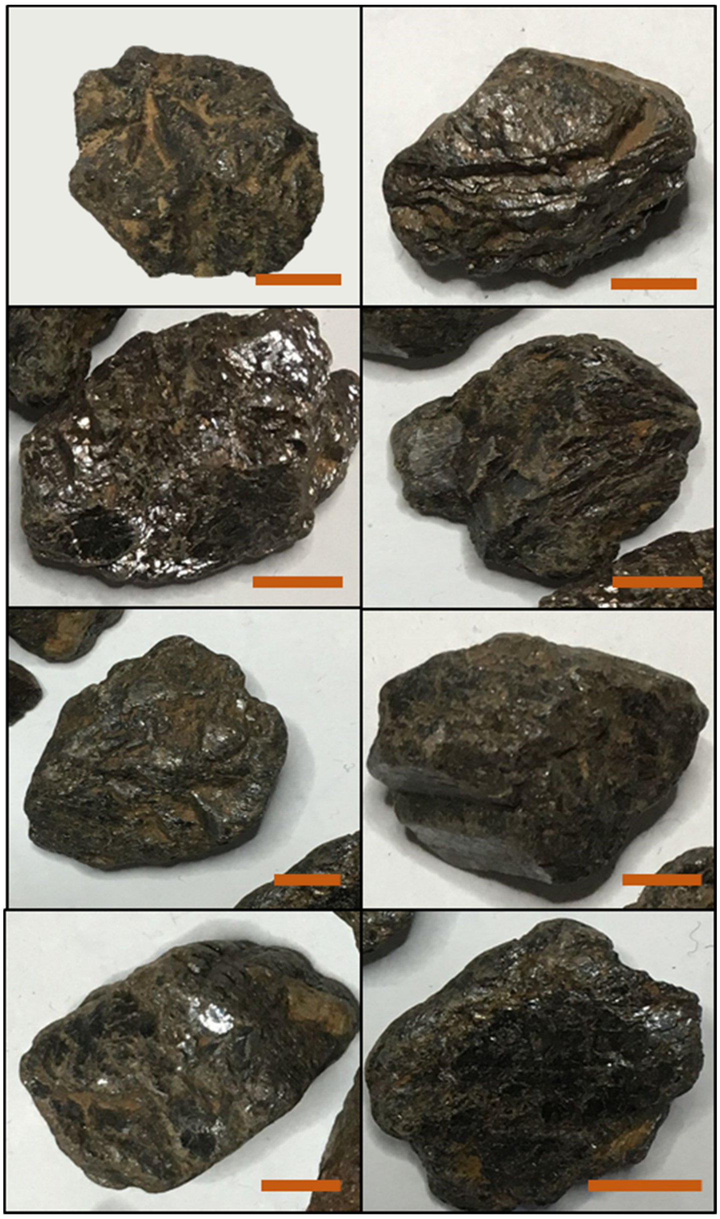

Figure S1. Photographs of the short prismatic rutile crystals from Brazil (the scale bar is 1 cm).

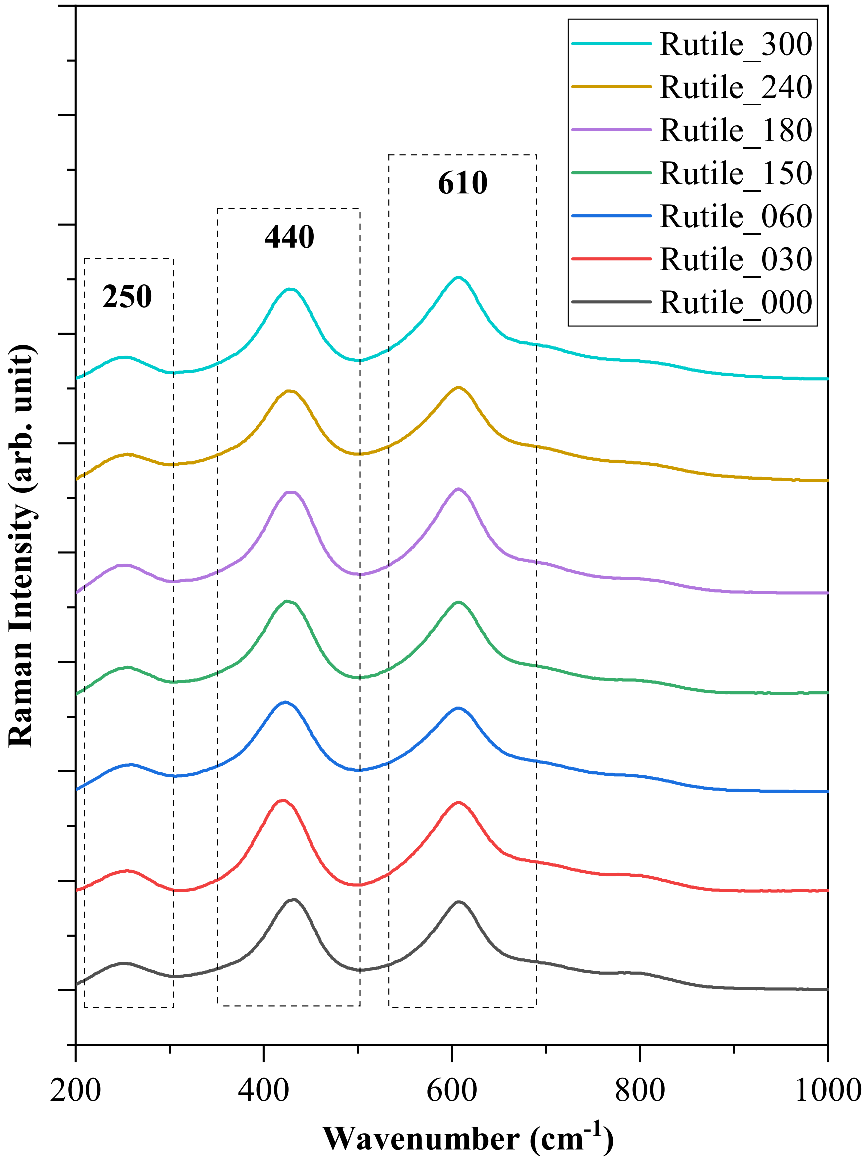

Figure S2. The Raman spectra of the grinded rutile, showing insignificant changes during the grinding process [53].

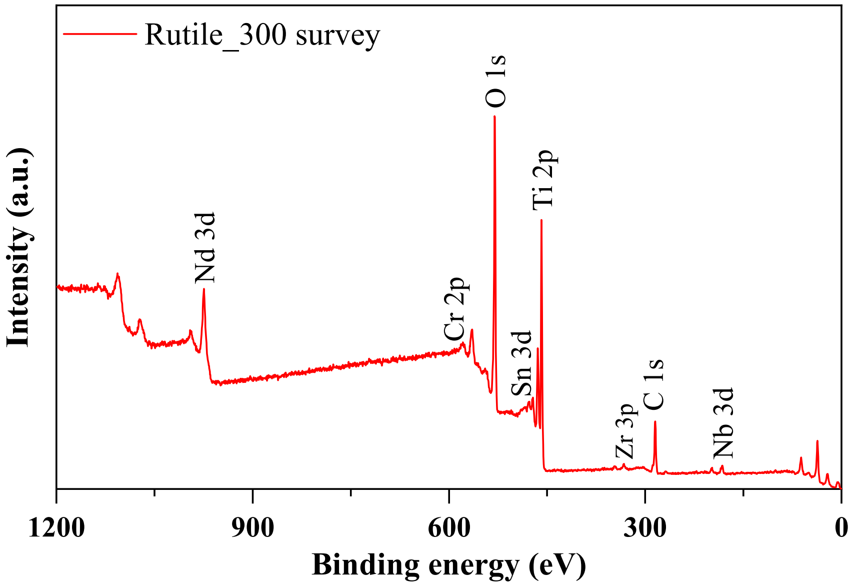

Figure S3. The XPS survey spectrum of the ground Rutile_300 sample.

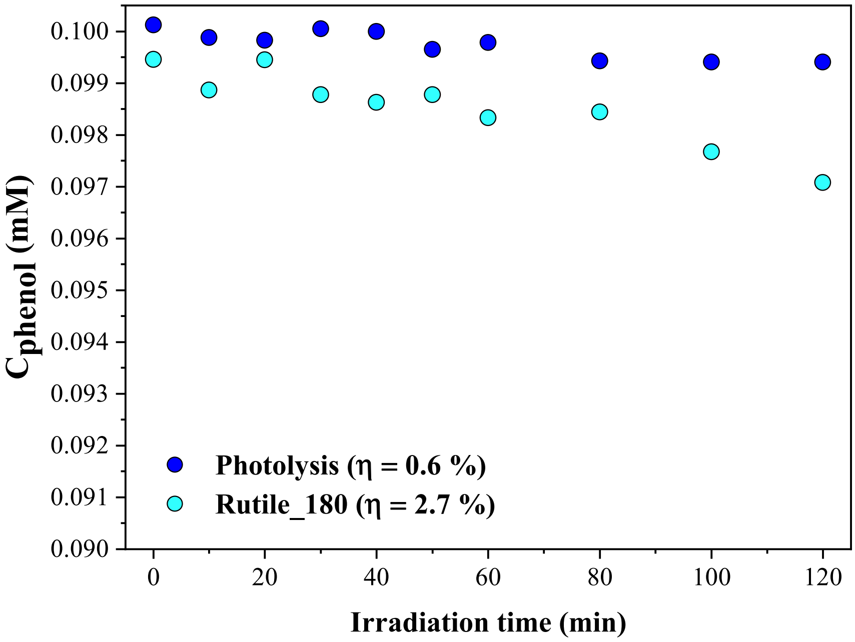

Figure S4. Phenol photolysis and photocatalytic degradation with Rutile_180 sample under UV light irradiation.

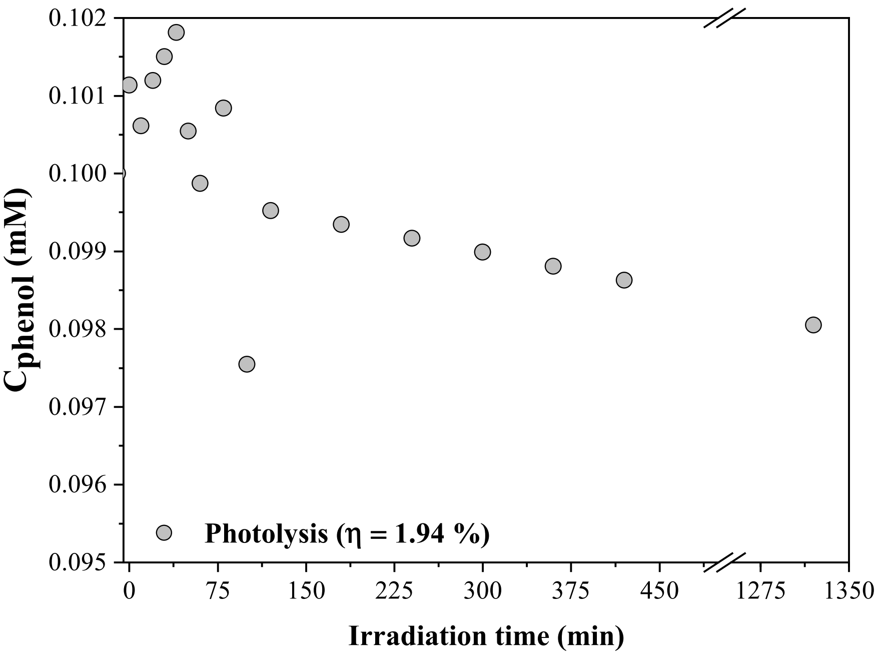

Figure S5. Phenol photolysis under visible light irradiation

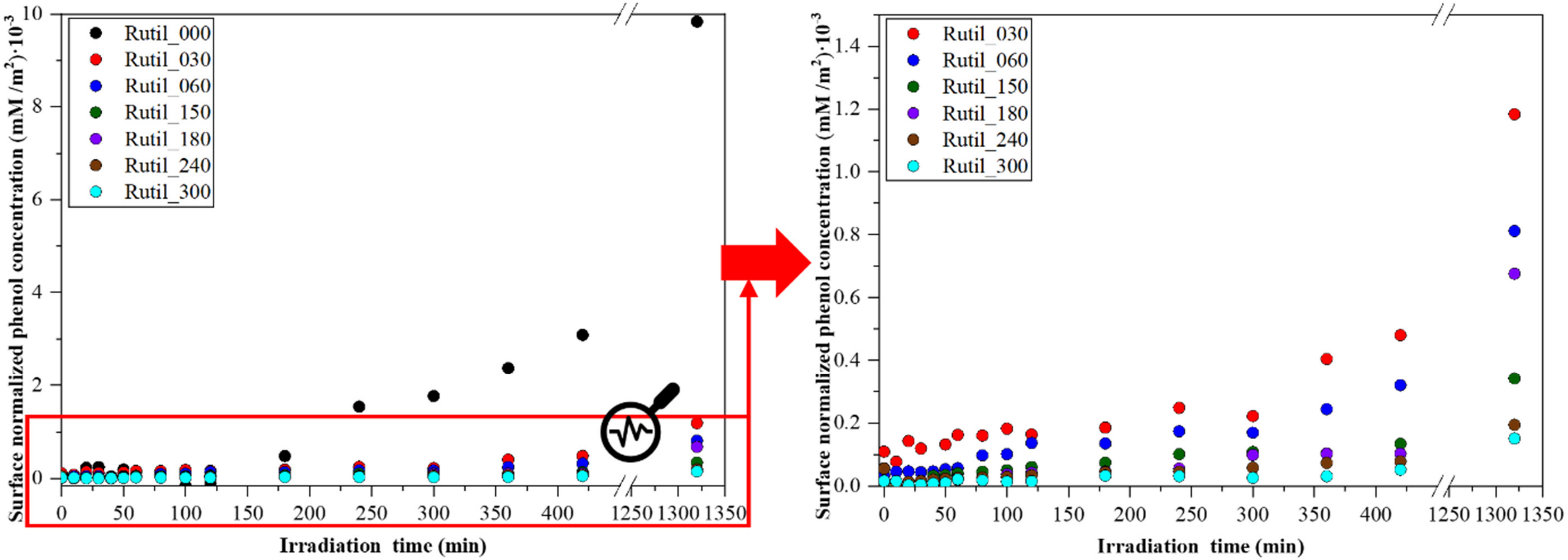

Figure S6. The surface normalized photocatalytic activity of grinded natural rutile samples for the degradation of phenol.

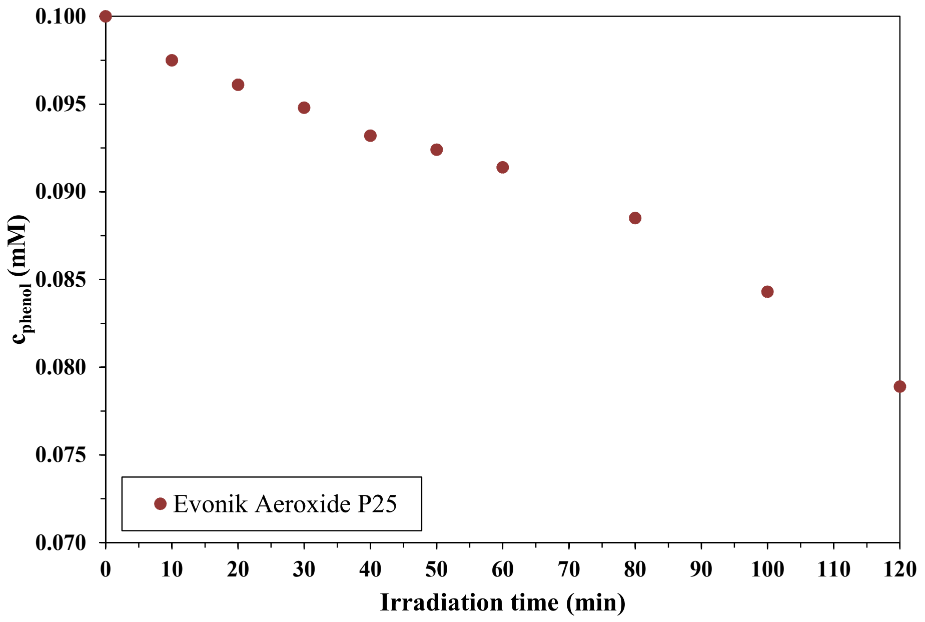

Figure S7. Photocatalytic degradation curve of phenol (c_{phenol}=0.1 mM) under visible light irradiation in the presence of commercial Evonik Aeroxide TiO_2 P25. The circumstances of the photodegradation test were the same as the ones described in *3.3 The photocatalytic activity determination* sub-section of the manuscript. The calculated degradation efficiency based on the above results is 21.1%.

2. Table

Table S1. Trace element concentration of grinded natural rutile samples XRF [53]/ XPS analysis

Trace elements concentration	Sample name			
	Rutil_180		Rutil_300	
	XRF (ppm)	XPS (at. %)	XRF (ppm)	XPS (at. %)
Ti	87.45 (%) *	n.a.	90.19 (%) *	19.6
O	n.a.	n.a.	n.a.	47.4
C	n.a.	n.a.	n.a.	29.4
Cr	0.0	n.a.	0.0	1.5
Cu	71	n.a.	64	0.0
Hf	4165	n.a.	1487	p.
Nb	2475	n.a.	506	p.
Nd	1092	n.a.	506	1.2
Ta	164	n.a.	106	p.
U	74	n.a.	54	0.0
W	230	n.a.	174	p.
Zn	48	n.a.	0	0.0
Zr	57630	n.a.	19210	0.5
Sn	0.0	n.a.	0.0	0.2
LOI (%)	6.71	n.a.	6.93	n.a

n.a. – not available, p.-present in the sample, but in very low concentration values with high error margins, LOI-Loss on ignition, * - quantity expressed as % of the total, due to high values

3. Cost-benefit calculations

3.1. *Cost estimation of the processing of natural rutile*

The price of high-quality rutile mineral ore gained after mining and beneficiation processes fluctuates depending on market trends, the grade of the material and the origin of the mineral. In the last years the cost typically ranged approximately from 1.15 to 1.5 €/kg [119]. For the sake of simplicity in the following calculations this raw material cost was considered, instead of the overall mining and beneficiation costs, and it was set to 1.5 €/kg.

An overall 5% material loss was considered for the milling process, meaning that for the production of 1 kg natural rutile powder there is a need of 1.05 kg raw material, which costs 1.575 €/kg.

3.1.1. *Calculation of costs of nano grinding of 1 kg of rutile in Netzsch MiniCer wet stirred media mill*

Available data from wet stirred media mill datasheet [120], utility prices [121, 122] or common knowledge:

- Batch volume (V_{total}): 500 mL
- Power of motor (P_{motor}): 0.94 kW

- Density of rutile (ρ_{rutil}): 4.25 g/mL
- Average cost of electricity in Romania ($Cost_{E\ Romania}$, 26 January 2025): 116 €/MWh
- Cost of water consumption in Romania, Cluj County ($Cost_{water\ Romania}$, 26 January 2025): 2.74 €/m^3

Defined circumstances:

- Filling ratio of grinding ball in the milling chamber ($R_{filling\ ball}$): 70 v/v%
- Solid content of slurry considered (R_{solid}): 40 m/m%
- Duration of grinding 1 batch ($t_{grinding}$): 180 min

Calculating electricity consumption of one batch:

$$t_{grinding} = \frac{180\ min}{60} = 3\ h \quad (1)$$

$$E_{batch} = P_{motor} \times t_{grinding} = 0.94\ kW\ \times 3\ h = 2.82\ kWh \quad (2)$$

Calculating the electricity costs per batch:

$$Cost_{E\ batch} = E_{batch} \times Cost_{E\ Romania} = 2.82\ kWh\ \times 0.116\ {}^{€}/_{kWh} = 0.32712\ € \quad (3)$$

Calculating water consumption of one batch:

$$R_{filling\ slurry}\ = 100 - R_{filling\ ball}\ = 30\ {}^{v}/_{v}\ \% \quad (4)$$

$$V_{slurry,batch} = V_{total} \times R_{filling\ slurry}\ = 500\ ml\ \times 0.3 = 150\ mL \quad (5)$$

$$V_{slurry,batch} = V_{rutil,batch} + V_{water,batch} = \frac{m_{rutil}}{\rho_{rutil}} + \frac{m_{water}}{\rho_{water}} = 150\ mL \quad (6)$$

$$R_{solid}\ = \frac{m_{rutil}}{m_{rutil}+m_{water}} \times 100 = 40\ {}^{m}/_{m}\ \% \quad (7)$$

Solving the above equations (6) and (7) we get: 86.45 g of rutile in a batch (appr. 20 ml in volume) and 130 ml water. Additionally, after each batch 2 liters of water were calculated for cleaning the milling chamber, adding up the water consumption to 2,13 liters/batch. Based on the here calculated batch size, the processing of 1 kg natural rutile can be done in 12 batches (12 batches would yield 1,037 kg rutile if we do not consider the material losses).

Calculating the water costs per batch:

$$Cost_{water\ batch} = V_{water,batch} \times Cost_{water\ Romania} = 2.13 \times 10^{-3}\ m^3\ \times 2.74\ {}^{€}/_{m^3} = 5.8\ \times 10^{-3}€ \quad (8)$$

Total cost/batch (86.45 g):

$$Cost_{total\ batch} = Cost_{E\ batch} + Cost_{water\ batch} = 327.12\ \times 10^{-3}\ €\ +\ 5.8\ \times 10^{-3}\ € = 332.92\ \times 10^{-3}€ \quad (9)$$

Total cost/kg of wet milling:

$$Cost_{total/kg} = \frac{Cost_{total\,batch} \times 1000}{m_{rutil,batch}} = \frac{332.92 \times 10^{-3} \times 1000}{86.45} = 3.85\ €/kg \quad (10)$$

If we consider material loss also: $Cost_{total} = Cost_{total/kg} \times 1.05 = 4.04\ €$ (11)

3.1.2. Calculation of costs of drying of 1 kg of rutile in a drying oven

Available data from drying oven *LabStac* OVE074-150 datasheet [123]and utility prices [121, 122]:

- Volume: 150 L
- Power 1.55 kW
- Average cost of electricity in Romania (26 January 2025): 116 €/MWh.

Defined circumstances:

- One drying cycle: 24 h
- Yield /cycle: capable of drying all slurry gained from grinding in one cycle (12 batches × $V_{slurry\ batch}$ = 1.8 L)

$$E_{drying} = P_{motor\ oven} \times t_{drying} = 1.55\ kW \times 24\ h = 37.2\ kWh \quad (12)$$

$$Cost_{E\ drying} = E_{drying} \times Cost_{E\ Romania} = 37.2\ kWh \times 0.116\ {}^{€}/_{kWh} = 4.31\ € \quad (13)$$

3.1.3. Estimated total cost of natural rutile ore processing

$$C_{rutile,total} = Cost_{raw} + Cost_{total\ grinding} + Cost_{E\ drying} = 1.575 + 4.04 + 4.31 = 9.93\ {}^{€}/_{kg} \quad (14)$$

3.2. Correlation of photocatalytic efficiency and product costs

Tabel S2. List of calculated data step by step to correlate the cost and the efficiency of the natural and synthetic photocatalyst

Sample	Data	Processed natural rutile	Evonik Aeroxide P25 TiO_2
Available data	Photocatalyst suspension concentration of phenol solution (mg/mL)	1	1
	Volume of phenol suspension (mL)	100	100
	Initial phenol concentration (mM)	0.1	0.1
	Irradiation time (h)	22	2
	Efficiency (%)	33.1	21.1
	Cost of photocatalyst (euro/kg)	9.93	265
Calculated	Cost of photocatalyst (euro/g)	0.0099	0.265
	Quant. of photocatalyst used (g)	0.1	0.1
	Quant. of phenol in total V (mmol)	0.01	0.01
	Quant. of degraded phenol (mmol)	0.0033	0.0021
	Quantity of degraded phenol/hour (mmol/h)	0.0002	0.0011
	Cost of photocatalyst used in the system (euro)	0.00099	0.0265
	Price of used photocatalyst/quantity of phenol degraded in an hour (euro/(mmol/h))	**6.6**	**25.12**

Abbreviations

$t_{grinding}$ – grinding time of one batch of natural rutile (h)
E_{batch} – electricity consumed for the grinding of one batch of natural rutile (kWh)
P_{motor} – power of the mill motor (kW)
$Cost_{E\ Romania}$ – cost of electricity in Romania (€)
$Cost_{E\ batch}$ – cost of electricity consumed for one grinding batch (€)
V_{total} – total milling chamber volume (mL)
$V_{slurry\ batch}$ – volume of slurry in one batch (mL)
$V_{rutil\ batch}$ – volume of rutil in one batch (mL)
$V_{water\ batch}$ – volume of water in one batch (mL)
$R_{filling\ ball}$ - filling ratio of grinding ball in the milling chamber (v/v%)
$R_{filling\ slurry}$ - filling ratio of slurry in the milling chamber (v/v%)
R_{solid} -solid content of slurry (m/m%)
$m_{water\ batch}$ – mass of water in one batch (g)
$m_{rutil\ batch}$ – mass of rutil in one batch (g)
ρ_{water} – density of water (g/mL)
ρ_{rutil} - density of rutil (g/mL)
$Cost_{water\ Romania}$ – cost of water in Romania (€)
$Cost_{water\ batch}$ - cost of water consumed for one grinding batch (€)
$Cost_{total\ batch}$ – total cost of grinding of one batch (€)
$Cost_{total/kg}$ – total cost of grinding/kg (€/kg)
$Cost_{total\ grinding}$ – total cost of grinding with material losses (€)
E_{drying} – electricity consumed for the drying of rutile (kWh)
$P_{motor\ oven}$ - power of the oven motor (kW)
t_{drying} - drying time of rutile (h)
$Cost_{E\ drying}$ - cost of electricity consumed for drying (€)
$Cost_{raw}$ - cost of raw material needed for the production of 1 kg of photocatalyst (€)
$Cost_{rutile\ total}$ – total cost of natural rutile photocatalyst production/kg (€/kg)

References of Supplementary Information

53. Almasi, E.; Pap, Z.; Racz, A.; Bohacs, K.; Mucsi, G.; Rakhely, G. Morpho-structural Properties of Nanogrinded Rutile as Potential Photocatalyst for Water Treatment. Geosciences and Engineering 2020, 8, 217-237.
120. Statista. Available Online: https://www.statista.com/statistics/1394503/global-price-of-titanium-minerals-by-type/ (last accessed on 01.02.2025).
121. Equip X -Netzsch Minicer Technical Data. Available Online: https://www.equipx.net/uploads/Netsch/NetzschMinicerTechnicalData.pdf (last accessed on 01.02.2025).
122. EU Energy. Available Online: https://euenergy.live/ (Last Accessed on 01.02.2025).
123. Somes Water Company S. A. Available Online: https://casomes.ro (last accessed on 01.02.2025).
124. Labstac Analytical & Lab Equipment, Drying Oven Data Sheet. Available Online: https://labstac.com/Catalog/Cp/OVE074-150/Drying-Oven-OVE074-150-Catalog-Labstac.pdf (last accessed on 01.02.2025).
